# Effects of an 11-week vibro-tactile stimulation treatment on voice symptoms in laryngeal dystonia

**DOI:** 10.3389/fneur.2024.1403050

**Published:** 2024-05-30

**Authors:** Jürgen Konczak, Divya Bhaskaran, Naveen Elangovan, Jinseok Oh, George S. Goding, Peter J. Watson

**Affiliations:** ^1^Human Sensorimotor Control Laboratory, School of Kinesiology and Center for Clinical Movement Science, University of Minnesota, Minneapolis, MN, United States; ^2^Department of Otolaryngology and Fairview Lion’s Voice Clinic, University of Minnesota, Minneapolis, MN, United States; ^3^Department of Speech, Language and Hearing Sciences, University of Minnesota, Minneapolis, MN, United States

**Keywords:** human, motor control, proprioception, somatosensory, spasmodic dysphonia

## Abstract

**Background:**

Laryngeal dystonia is a task-specific focal dystonia of laryngeal muscles that impairs speech and voice production. At present, there is no cure for LD. The most common therapeutic option for patients with LD involves Botulinum neurotoxin injections.

**Objective:**

Provide empirical evidence that non-invasive vibro-tactile stimulation (VTS) of the skin over the voice box can provide symptom relief to those affected by LD.

**Methods:**

Single-group 11-week randomized controlled trial with a crossover between two dosages (20 min of VTS once or 3 times per week) self-administered in-home in two 4-week blocks. Acute effects of VTS on voice and speech were assessed in-lab at weeks 1, 6 and 11. Participants were randomized to receive either 40 Hz or 100 Hz VTS.

**Main outcome measures:**

Primary: *smoothed cepstral peak prominence* (CPPS) of the voice signal to quantify voice and speech abnormalities, and *perceived speech effort* (PSE) ranked by participants as a measure of voice effort (scale 1–10). Secondary: *number of voice breaks* during continuous speech, the *Consensus Auditory-Perceptual Evaluation of Voice* (CAPE-V) inventory as a measure of overall disease severity and the *Voice Handicap Index* 30-item self report.

**Results:**

Thirty-nine people with a confirmed diagnosis of adductor-type LD (mean [SD] age, 60.3 [11.3] years; 18 women and 21 men) completed the study. A single application of VTS improved voice quality (median CPPS increase: 0.41 dB, 95% CI [0.20, 0.61]) and/or reduced voice effort (PSE) by at least 30% in up to 57% of participants across the three study visits. Effects lasted from less than 30 min to several days. There was no effect of dosage and no evidence that the acute therapeutic effects of VTS increased or decreased longitudinally over the 11-week study period. Both 100 and 40 Hz VTS induced measurable improvements in voice quality and speech effort. VTS induced an additional benefit to those receiving Botulinum toxin. Participants, not receiving Botulinum treatment also responded to VTS.

**Conclusion:**

This study provides the first systematic empirical evidence that the prolonged use of laryngeal VTS can induce repeatable acute improvements in voice quality and reductions of voice effort in LD.

**Clinical trial registration:**

ClinicalTrials.gov ID: NCT03746509.

## Introduction

Laryngeal dystonia (LD) – also called spasmodic dysphonia – is a task- specific focal dystonia of laryngeal muscles that impairs speech and voice production (1). About 80% of patients present with adductor type LD, where spasms of laryngeal adductor muscles force the vocal folds to close, leading to a strangled, strained speech with uncontrolled voice breaks (2). In contrast, abductor type LD is characterized by hyperabduction and uncontrolled vocal fold opening resulting in a “breathy” or whispery voice. At present, there are limited therapeutic options for patients with LD but no cure. The most common treatments are regular Botulinum neurotoxin (BoNT) injections into the laryngeal muscles which typically provides temporary symptom relief for 2–5 months (3). However, efficacy is variable with only 50% of LD patients receiving benefits from BoNT injections (4).

The pathophysiology of LD is incompletely understood. There is consensus that it involves structural and functional changes within a network comprising basal ganglia, cerebellum, sensorimotor cortex and brainstem (5–9). This is consistent with findings of empirical studies of other forms of focal dystonia. For example, applying transcranial magnetic stimulation to people with focal hand dystonia (i.e., writer’s cramp) revealed a loss of inhibition at the cortex (10, 11) and altered functional connectivity between cortical–subcortical sensorimotor regions in cervical dystonia (12). Recent evidence shows that LD is characterized by a reduced movement-related desynchronization over laryngeal somatosensory-motor cortex (13) – a phenomenon also observed in cervical dystonia (14, 15) and writer’s cramp (16).

Importantly, LD and other forms of focal dystonia are associated with tactile and proprioceptive dysfunction (17). This raises the question, if a targeted neuromodulation of the somatosensory system such as vibro-tactile stimulation (VTS) of the skin above the larynx could alleviate the speech symptoms of LD. It has long been established that VTS between 40 and 100 Hz can stimulate muscle spindles (18, 19) and cutaneous mechanoreceptors (20) affecting motor behavior and inducing changes in kinaesthesia (21, 22). With respect to LD, previous work demonstrated that short applications of VTS (< 30 min) induced fast improvements in voice quality in over two-thirds of the participants. The neural correlate of such behavioral improvement is that VTS normalizes the abnormally high levels of synchronization of sensorimotor cortical neurons associated with LD symptoms (13). This VTS-induced electrocortical effect is similar in nature to what is observable in people with cervical dystonia who apply sensory tricks (23) suggesting that effectiveness of VTS and sensory tricks may share a similar neural mechanism. With respect to other forms of focal dystonia, a case study (24) reported that VTS applied to trapezius muscles normalized the head posture of a patient with torticollis and neck muscle VTS may reduce pain associated with cervical dystonia (25). In summary, previous research established proof-of-concept of the short-term effectiveness of VTS in treating the symptoms of focal dystonia and provided insights into the underlying neural mechanism behind its effectiveness. However, insights into its longer-term effect over days and months are missing, which would be important to establish its clinical usefulness for LD.

To address this question, this study systematically examined the longitudinal effect of VTS over a period of 11 weeks, in which people with LD applied VTS at home. Specifically, they received VTS at either 40 Hz or 100 Hz where the lower frequency condition only activated tactile mechanoreceptors of the skin which mimics a sensory trick, while a 100 Hz stimulation would also trigger responses of mechanoreceptors in laryngeal muscles, the mucosa of the epiglottis and sensory corpuscles at the free edge of the vocal cords (26, 27).

## Materials and methods

### Participants

Participants with adductor-type LD were recruited from the Lions Voice Clinic at the University of Minnesota as well as through U.S.-wide announcements made by the National Spasmodic Dysphonia Association. The inclusion criterion for participation was a confirmed diagnosis of adductor-type LD. Exclusion criteria were (1) abductor type of LD, (2) other concurrent signs of focal dystonia, and/or (3) other neurological signs that could affect voice or speech production. The study was approved by the Institutional Review Board of the University of Minnesota and registered with clinicaltrials.gov (*Study identifier*: *NCT03746509*). LD was diagnosed clinically by otolaryngologists, based primarily on history, voice, and laryngoscopic findings. As part of the examination, all patients at the University of Minnesota completed a battery of vocal tasks with trained speech pathologists and a laryngeal exam conducted by an otolaryngologist.

All participants gave written informed consent prior to study begin. To control for the effect of BoNT injections on voice quality improvement, the first study visit of BoNT-treated participants was scheduled 2–3 weeks after their last injection assuring that symptoms during the first study visit were not influenced by the recent injection (e.g., a period of weak and breathy voice).

### Equipment

**Figure 1:**
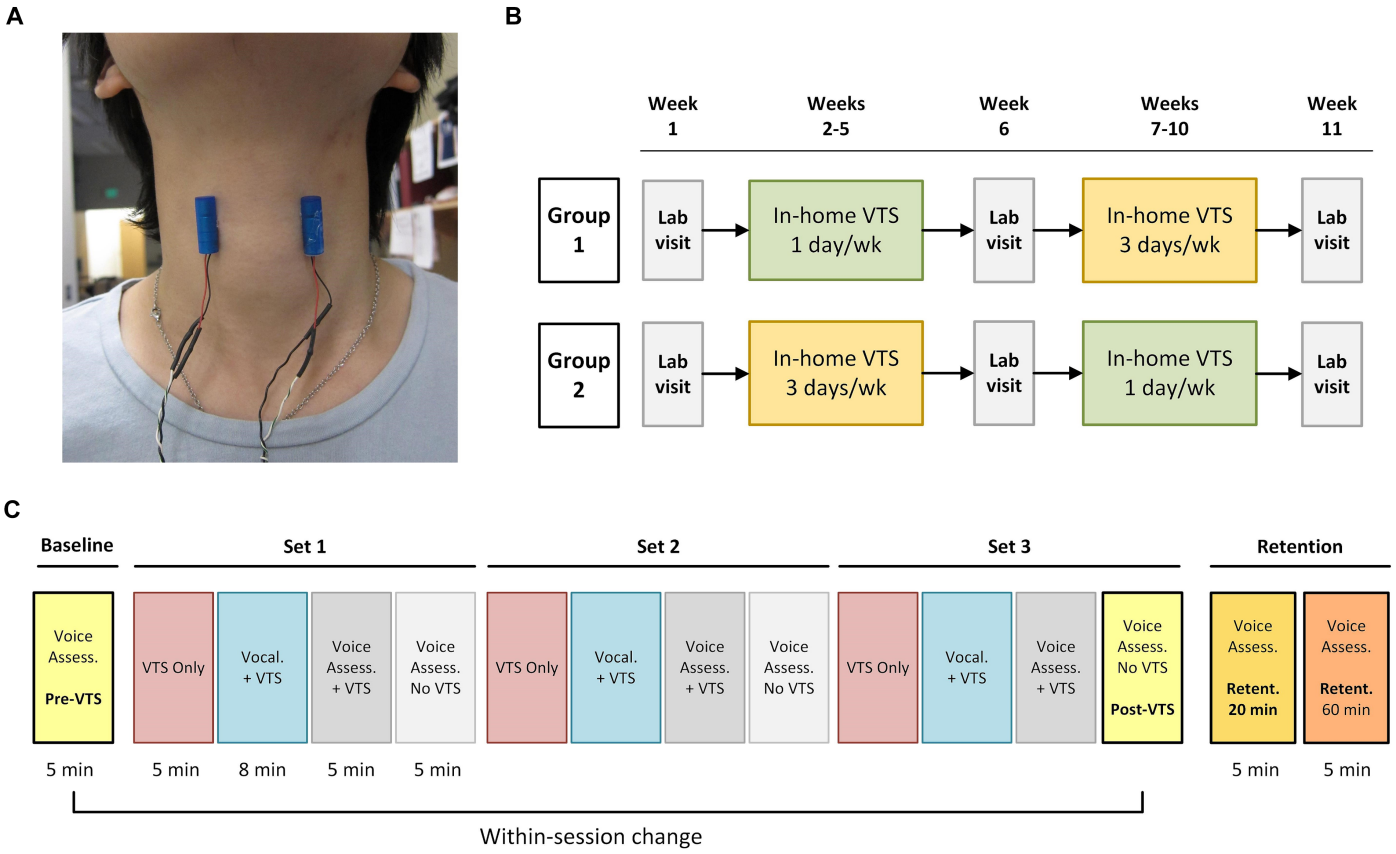
Experimental design and procedure during study visits. **(A)** Placement of the encapsulated vibrators on the skin above the larynx at the level of the hyoid bone (vibrator diameter: 9 mm, length 25 mm). **(B)** The study employed a crossover experimental design. Participants visited the lab at week 1, 6, and 11. One group started in-home VTS with a dosage of 20 min for 1 day/week, while the other group received 3 days/week VTS. Groups crossed over at week 7. Participants in each group were randomly assigned to receive VTS at either 40 Hz or 100 Hz (no crossover of frequency). **(C)** Process chart of the in-lab visit procedure. During the *VTS Only* condition, vibrators were turned on for 5 min while the participant sat silently. The *Vocalization + VTS* condition consisted of 50 trials. In each trial, participants received an auditory cue (1,000 Hz, 98 dB) for 250 ms and then vocalized the vowel /a/ continuously for 4 s. After 2 s of vocalization, laryngeal VTS was applied for the remaining 2 s. A 4-s-long resting interval followed each trial. Participants received three sets of 18 min VTS for a total of 54 min. Retention was assessed 20 and 60 min past the *Post-VTS* time point.

Vibro-tactile stimulation was administered via a pair of lightweight encapsulated vibro-motors (Model 307–100, Precision Microdrives Ltd., London, UK). The vibro-motors were attached bilaterally over the thyroid cartilage lamina (Figure 1A). Our previous work determined that a vibration frequency of 100 Hz at the skin results in 60–70 Hz vibration at the larynx, which is within the frequency range known to stimulate laryngeal mechanoreceptors (26). Superficial stimulation at 40 Hz is too low to stimulate laryngeal mechanoreceptors. However, it does stimulate tactile mechanoreceptors such as Meissner corpuscles in the skin over the thyroid cartilage, which respond to low-frequency vibrations (30–50 Hz). All participants received a set of vibrators with power supply and were trained on the in-home use of VTS.

### Study design

The 11-week study followed a longitudinal crossover randomized controlled experimental design. It included three in-lab visits for assessments at weeks 1, 6 and 11 and two blocks of 4-week in-home treatment where participants self-administered VTS (see Figure 1B). Participants were randomly assigned to either a *low* (20 min. VTS at 1 day/week; *n* = 20) or *high* dosage (20 min. VTS at 3 days/week; *n* = 19) treatment for the first block at weeks 2–5 and then crossed over to the second treatment block (weeks 7–10). For example, a participant completed an at-home low-dosage regimen in weeks 2–5 and crossed over to an at-home high-dosage regimen in weeks 7–10. Within each group, participants were randomly assigned to receive VTS of either 100 Hz or 40 Hz. Experimenter NE generated the random allocation sequence, and DB enrolled and assigned participants to interventions. The multiple baseline study design addressed the potential cofound of BoNT administration as each study visit established a new baseline and compared the effect of VTS with respect to that baseline. That is, reported effects of VTS represent a change in addition to the symptom decreasing effect of BoNT in those participants who received BoNT.

### Study visit procedure

In-lab testing took place in a specialized facility. Participants sat in an electrically and acoustically shielded chamber (2.14 × 2.14 × 1.98 m; 60–70 dB attenuation of outside sound; ETS-Lindgren Acoustic Systems, Cedar Park, TX, USA) to assure that all voice signals were not contaminated by outside noise. For voice recordings, a professional recording system (Marantz CDR 300 recorder and AKG C420 microphone) was used with the microphone being placed approx. 7–10 cm from the participant’s mouth. A pair of vibro-motors was attached to the skin over the laryngeal area (Figure 1B). The protocol started with a baseline voice assessment. Then participants received three sets of VTS that each began with a *VTS Only* condition (vibrators were turned on for 5 min), followed by a *Vocalization + VTS* condition, in which they vocalized the vowel /a/ while VTS was on, and concluded with two voice assessments with and without VTS. Retention of possible VTS effects were assessed 20 and 60 min (Ret-20, Ret-60) after the cessation of VTS (Figure 1C). Each voice assessment consisted of participants (1) reading aloud a series of standard SD symptom-eliciting sentences (2) and (2) rating voice effort while speaking test sentences at their habitual pitch and loudness. At study end, participants completed a structured exit interview.

### Measurements

Audio recordings were anonymized by experimenter D.B. and sent to two speech-pathology experts, who were blinded to the randomization, dosage allocation and study visit time points. The following measures were obtained from the audio signals using PRAAT software (28).

### Primary outcome measures

*Smoothed cepstral peak prominence (CPPS)*. This is an established objective measure of voice quality to quantify abnormalities of the voice signal in speech (29) that correlates well with perceptual judgments of dysphonia severity (30, 31). Cepstral analysis has been shown to correlate with the severity of voice impairment in patients before and after BoNT therapy (32).

*Perceived speech effort* (PSE) rated by participants on a scale of 0–10 (10 indicates maximum vocal effort) (33).

The absolute change of either outcome measure was computed as the difference between *Pre-VTS* and the later time points (*Post-VTS*, *Ret-20*, *Ret-60*) and as ΔCPPS or ΔPSE. Relative change was denoted as rCPPS or rPSE and calculated as:

$\begin{array}{l} \mathrm{Relative}\;\mathrm{Change}=\left[\left(\mathrm{PostVTS}-\mathrm{PreVTS}\right)÷\mathrm{PreVTS}\right]\\ \;\times 100\;\left(\mathrm{unit}:\%\right)\; \end{array}$ (1)

### Secondary outcome measures

The *number of voice breaks* during continuous speech – a symptom of adductor-type LD (2).

The *Consensus Auditory-Perceptual Evaluation of Voice* (CAPE-V) inventory (scale of 0–100 with 100 indicating severe dysphonia) (34). CAPE-V ratings at week 1 indicated symptom severity prior to intervention (score between 30 and 65 = moderate symptoms, 65–100 = severe symptoms). A speech-language pathologist with over 10 years of clinical voice experience independently determined overall disease severity.

The *Voice Handicap Index* (VHI) is a self-rated 30-item inventory to indicate the impact of experienced voice problems or a voice disorder (score range: 0–120, score between 0 and 30 = mild severity; 31–60 = moderate severity; 61–120 = severe severity) (35).

### Statistical data analysis

Shapiro–Wilk tests with Bonferroni adjustment were performed for all outcome measures to test for the assumption of normality. The distribution of CPPS, CAPE-V, PSE and rCPPS, rPSE departed from normality for at least one time point within (*Pre-VTS, Post-VTS, Ret-20, Ret-60*) or between lab visits (*week 1, 6, 11*). Consequently, non-parametric Wilcoxon-signed-rank tests were conducted to examine the time-dependent and dosage-related effects of VTS. Respective 95% confidence intervals (CI) were computed using the percentile bootstrap method (36). The Rosenthal correlation coefficient (r) was calculated as a measure of effect size. Based on previous data (37), an *a priori* power analysis (*α* = 0.05, power = 0.95) using ΔCPPS showed that *n* = 32 participants (*n* = 16 per crossover dosage group) were required to yield a significant pre- to post-VTS difference. This report follows the CONSORT reporting guidelines (38).

## Results

A total of 39 patients with adductor-type LD participated and completed the study (mean age: 60 yrs. SD ± 11.3; 18 females, 21 males) and their data were included in the analysis (see Table 1). Four participants completed the study protocol twice. After a 6-week wash-out period, they repeated the protocol and switched to the VTS frequency they had not received before. Three participants had to stop the trial due to COVID-19 related travel restrictions. Data collection occurred from 30 April 2019 to 23 May 2023. Recruitment stopped on 24 May 2023. Analysis of the reported data was completed by 2 Jan 2024.

**Table 1:** Demographics and clinical presentation of the 39 participants.

No.	Subj ID	Sex	Age (years)	Symptom duration (years)	Symptom severity (CAPE-V) at baseline	Voice Handicap Index (VHI) at baseline	Presence of vocal tremor	BoNT therapy	Last BoNT prior study begin (weeks)	BoNT injection cycle (months)
1	01	M	57	10	85	97	No	Yes	10	3
2	03	M	70	32	70	49	Yes	No	39	6
3	04	F	79	12	70	32	No	Yes	13	2.5–3
4	05	M	50	8	40	71	No	No	26	18
5	06	M	65	39	80	28	No	Yes	2	1–2
6	09	F	61	8	N/A	37	No	Yes	2	3–4
7	10	F	59	4	98	81	Yes	No	n/a	n/a
8	11	M	52	9	90	62	Yes	Yes	2	3–4
9	12	M	66	35	70	58	No	Yes	2	4–5
10	13	M	54	14	40	62	No	No	104	6–12
11	15	F	66	8	60	81	No	No	52	3–4
12	16	F	62	13	80	76	Yes	No	n/a	
13	17	M	70	10	40	46	Yes	Yes	2	3
14	18	M	54	13	70	60	No	Yes	2	3–4
15	19	F	70	13	70	66	No	No	156	
16	20	F	75	9	50	57	No	Yes	3	3
17	21	F	54	8	70	59	No	Yes	2	2–3
18	23	M	56	5	75	88	No	Yes	2	3
19	24	F	58	4	60	91	No	Yes	2	1.5
20	25	M	29	4	60	57	No	Yes	2	3–5
21	26	F	60	10	95	89	No	Yes	2	6
22	27	F	59	14	70	68	No	Yes	2	2
23	28	F	33	5	60	95	No	Yes	2	3
24	29	F	49	2	60	63	No	Yes	2	3
25	30	F	53	13	45	64	No	Yes	2	3–4
26	31	M	71	31	85	55	Yes	No	52	24
27	32	F	69	55	65	73	Yes	Yes	2	2
28	33	M	68	6	45	64	No	Yes	2	6
29	34	F	67	11	60	89	Yes	No	417	5
30	35	M	35	9	85	81	No	No	26	n/a
31	36	F	68	3	50	49	Yes	Yes	3	3
32	37	M	66	30	40	66	No	Yes	2	5
33	39	F	65	12	70	55	Yes	No	110	n/a
34	40	M	75	12	75	33	No	No	282	n/a
35	41	F	67	7	90	101	Yes	No	n/a	n/a

33 participants completed all three study visits over the 11-week study duration and 6 participants completed only two study visits due to COVID-19 pandemic-related travel restrictions. Symptom severity is indicated by the CAPE-V (score 0–100; higher score indicates higher symptom severity) and the Voice Handicap Index (score 0–120; higher score indicates higher symptom severity) at the week 1 baseline prior to receiving any VTS. CAPE-V was rated by a voice disorder specialist, VHI was self-rated by the participant. n/a: never received BoNT treatment.

### Acute and longitudinal effects of VTS on voice quality and speech effort

To determine short-term effect of VTS within a study visit, outcome measures at Post-VTS and the two retention time points were compared relative to Pre-VTS.

### Primary outcome measure

The median absolute change in CPPS (ΔCPPS) at Post-VTS compared to Pre-VTS was significantly different from zero (med: 0.41 dB, 95% CI [0.20, 0.61], *r* = 0.35). However, median ΔCPPS was not significantly different from zero at the two retention time points (med at *Ret-20*: 0.11 dB, 95% CI [−0.08, 0.30], *r* = 0.11; med at *Ret-60*: 0.2 dB, 95% CI [−0.00002, 0.39], *r* = 0.18). Median absolute change in speech effort was significantly smaller at Post-VTS (med ΔPSE: −1.0, 95% CI [−1.5, −0.99], *r* = 0.48) and at both retention time points (med at *Ret-20*: −1.0, 95% CI [−1.5, −0.99]; *r* = 0.44; med at *Ret-60*: 0.0, 95% CI [−1.5, −0.99], *r* = 0.44).

### Secondary outcome measures

Two-third of the participants (26/39) presented with voice breaks in at least one of the study visits. Of those, six participants (23%) reduced their number of voice breaks at Post-VTS in week 1 (median reduction: 2.5; range: 1–24), four participants (15%) in week 6 (median reduction: 6.5; range: 1–23), and nine participants (35%) in week 11 (median reduction: 4; range: 1–12).

**Figure 2:**
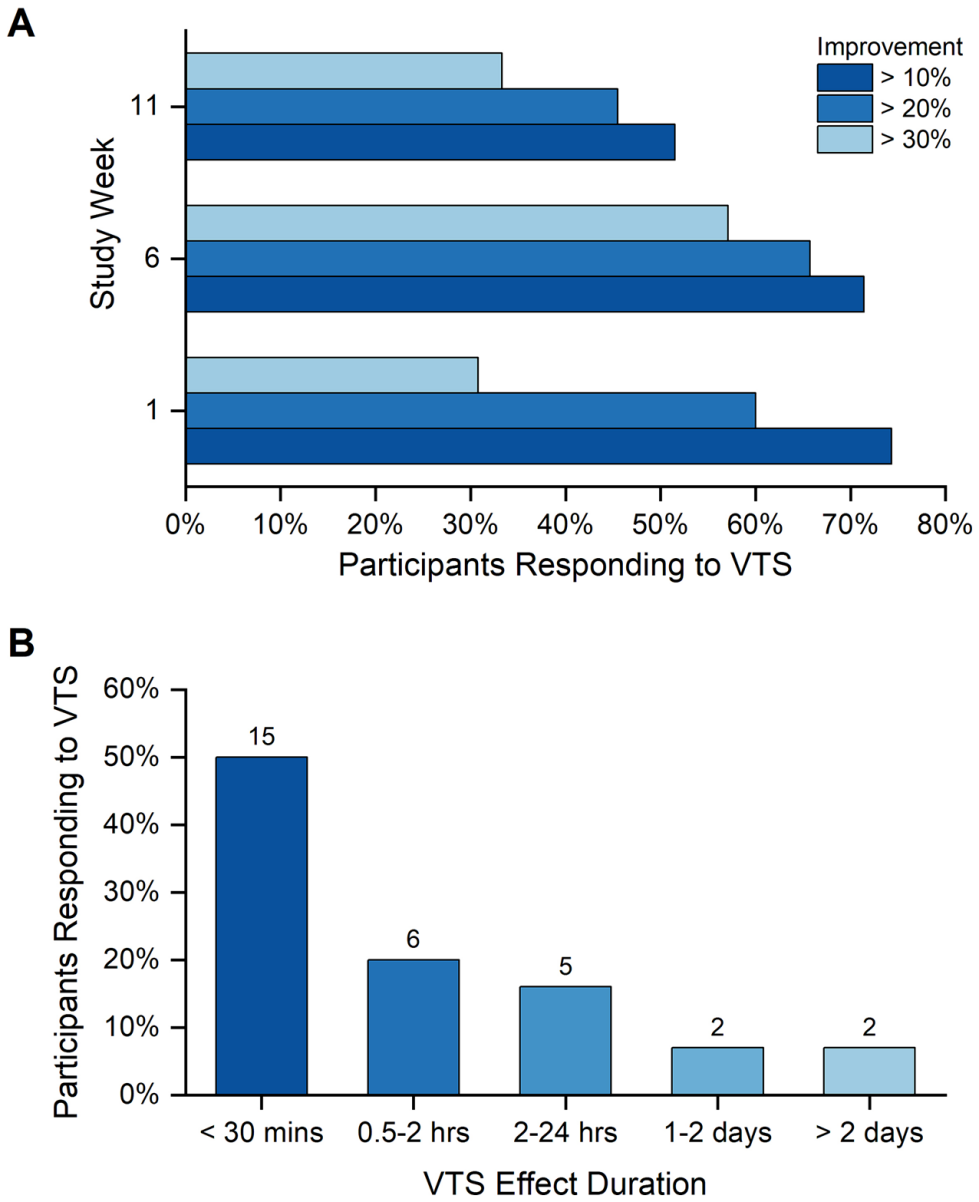
Participant response rate to VTS. **(A)** Shown are the response rates to VTS across the study visits at weeks 1, 6, and 11. Bars indicate the rate of participants responding to VTS at levels of at least 10, 20% or 30% improvement in one or both outcome measures (i.e., a respective increase in CPPS and/or decrease in PSE). Improvement was measured as the change between *Pre-VTS* and *Post-VTS*. **(B)** The duration of VTS effectiveness as perceived by study participants at study end. Data reflect the responses of 30 participants to the question “how long did you feel the effect of VTS.”

Over the three study visits, between 31 and 57% of participants exhibited a relative improvement of at least 30% (i.e., a respective increase in CPPS and/or decrease in PSE; Figure 2A). With respect to the VTS effect duration, responses from 30 participant exit interviews indicated that for 15 (50%) of participants the acute effect lasted less than 30 min, for 6 (20%) the effect had decayed within 2 h, and 9 (30%) participants reported that the beneficial effect lasted more than 1 day (Figure 2B).

**Figure 3:**
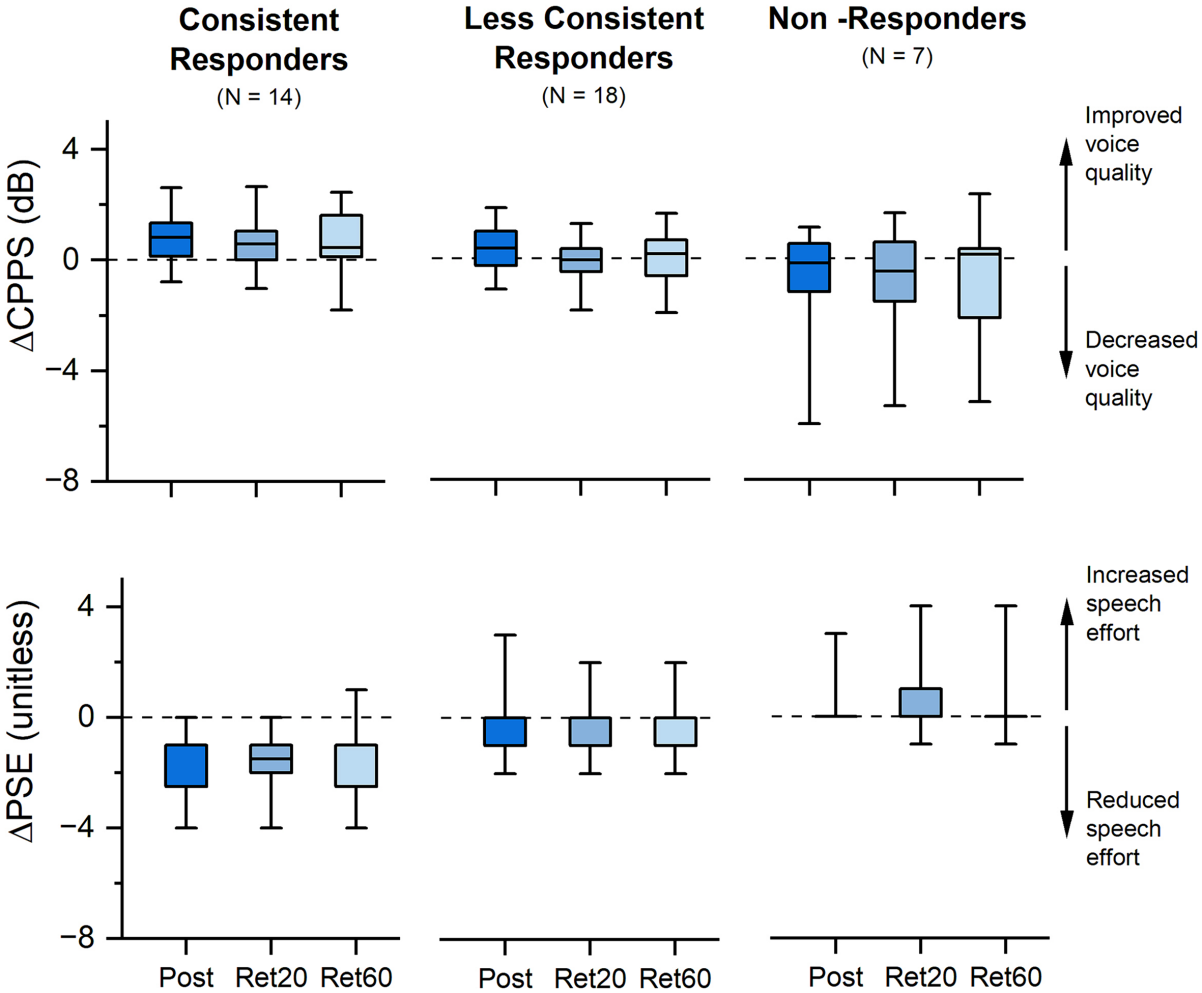
Effect of VTS on voice quality and speech effort. Shown is the absolute change in CPPS and PSE at *Post-VTS*, 20 min (*Ret 20*) and 60 min (*Ret 60*) relative to *Pre-VTS* for all three study visits. *Consistent responders* exhibited a reduction in PSE and/or an increase in CPPS of 10% or more in all three study visits (week 1, 6, 11), and *less consistent responders* in one or two visits. *Non-responders* did not show a reduction in PSE and/or an increase in CPPS in any of the three study visits. Boundaries of each box represent the 25th and 75th percentile. Line within the box represents the median. Whiskers represent the 5th and 95th percentile.

Considering a relative improvement of at least 10% in either CPPS or PSE as a response threshold, 46% (18/39) responded to VTS in one or two sessions, and 36% (14/39) showed a consistent response in all three study visits. Consistent responders tended to exhibit positive change in both outcome measures that persisted at the two retention time points (Figure 3).

### No differential effect of weekly VTS dosage on voice outcome measures

During the in-home phases of the study, participants received 20 min of VTS once or three times per week. Median differences between both dosage conditions (3x/wk = high; 1x/wk = low) were not significantly different for rCPPS at the end of the first treatment block (high/low difference: 2.0, 95% CI [−5.5, 9.0], *r* = 0.12) and the second block (high/low difference: −8.5, 95% CI [−17.3, −2.1], *r* = 0.4) as well as for rPSE after the first block (high/low difference: 0.0, 95% CI [−25, 25], *r* = 0.02) and second block (high/low difference: 0.0, 95% CI [−20, 37], *r* = 0.13).

### No differential effect of VTS frequency on voice outcome measures

**Figure 4:**
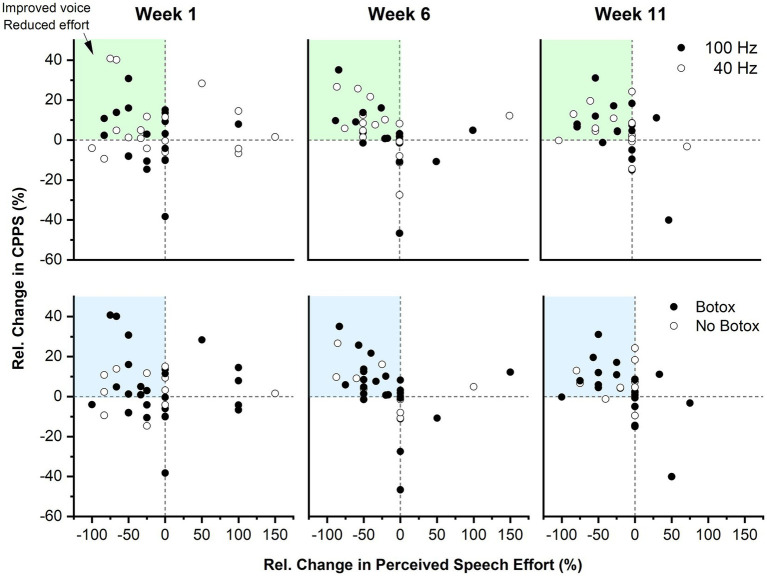
Longitudinal effect of VTS on the relative change in CPPS and PSE as a function of stimulation frequency and BoNT. Top panel: Each data point represents the *Pre-VTS* to *Post-VTS* change within a study visit of a single participant. The dark blue shaded region indicates the quadrant where participants exhibited combined improvements in CPPS as well as PSE. The light blue shaded quadrants reflect regions where participants exhibited positive change in one of the two outcome measures. Data are split into two groups receiving either 40 Hz or 100 Hz VTS. Bottom panel: The same data as in the top panel are shown, but categorized by those who received BoNT injections and those who did not.

Absolute change in CPPS yielded no statistically significant differences in median between 40 Hz and 100 Hz VTS at either time point (*week 1* difference: 0.21 dB, 95% CI [−0.67, 0.93], *r* = 0.09; *week 6* difference: 0.37 dB, 95% CI [−0.24, 1.03], *r* = 0.2; *week 11* difference: −0.17 dB, 95% CI [−0.68, 0.65], *r* = 0.07). Absolute change in PSE yielded no statistically significant differences in median between 40 Hz and 100 Hz VTS at either time point (*week 1* difference: 0, 95% CI [−1, 1], *r* = 0.03; *week 6* difference = 0, 95% CI [−1, 1], *r* = 0.08; *week 11* difference: 0, 95% CI [−1, 1], *r* = 0.01). To obtain an understanding on the longitudinal change in both primary outcome measures, the top panel of Figure 4 shows the relative change in CPPS and PSE by stimulation frequency for all participants across the three study visits.

### Laryngeal VTS can provide additional benefits to those treated with botulinum neurotoxin

An ancillary analysis examined if potential benefits of VTS are explained as an effect of concurrent Botulinum neurotoxin treatment. A total of 26 participants were regularly treated with BoNT injections and 24 had received their last injection between 2 and 3 weeks before study begin (see Supplementary material). Analysis of the aggregate data across the three study visits yielded no significant difference in ΔCPPS between participants who received BoNT versus those who did not (*week 1* difference: 0.00 dB [−0.80, 1.00], *r* = 0.0; *week 6* difference: −0.18 dB, 95% CI [−0.89, 0.57], *r* = 0.08; *week 11* difference: 0.23 dB, 95% CI [−0.44, 0.94], *r* = 0.09).

Among those participants who received BoNT, 14 participants were on a 3-months cycle. This population typically experiences the most benefits from BoNT around halfway through the cycle (i.e., 6 weeks) and then a reduction in voice quality toward the end of their cycle. An analysis of these 14 participants showed that 74% of them had an improvement of 10% or more in CPPS or PSE due to VTS at week 6 and 55% still maintained these benefits at week 11. This implies that VTS provided an additional benefit to those treated with BoNT. The bottom panel of Figure 4 shows the relative change in CPPS and PSE by BoNT or no BoNT injection for all participants across the three study visits.

## Discussion

This is the first clinical trial to systematically examine the acute and longitudinal effects of applying non-invasive vibro-tactile stimulation on the skin above the larynx of people with adductor-type laryngeal dystonia.

### Acute and longitudinal effects of VTS on voice quality and speech effort

Depending on the outcome measure, laryngeal VTS induced meaningful acute changes in speech effort and voice quality in approximately one-third to one-half of the participants who exhibited a 30% or higher improvement in one or both outcome measures (see Figure 2). Empirical evidence from this study and our earlier work (37) shows that the effects occur within 15–20 min of stimulation. These findings align with data on the neurophysiological mechanism behind the effectiveness of VTS in treating the voice symptoms of LD. The electrocortical response to VTS is measurable as a desynchronization of motor cortical neuron activity that normalizes the abnormally high levels of synchronization of sensorimotor cortical neurons observed in LD (13).

After the cessation of VTS, the effects of VTS on CPPS as a marker of voice quality tended to decay fast within 30 min, while effects on perceived speech effort were still significantly different from baseline after 60 min (see Figure 3). Subjective impression reports revealed that a third of the participants reported symptom reduction for up to a day, and 14% indicated that effects of one-time VTS can last several days (see Figure 2B). We found no conclusive evidence that possible effects of VTS built up longitudinally over the time course of weeks or months as participants did not systematically exhibit higher or lower rates of improvement in both outcome measures at the end of the trial.

### Effect of VTS stimulation frequency on voice quality and speech effort

In this study participants received laryngeal VTS at stimulation frequencies of 40 or 100 Hz. The rationale for selecting these frequencies arose from the lack of knowledge, if the assumed effectiveness of VTS required the activation of proprioceptive mechanoreceptors of the larynx or if the stimulation of tactile mechanoreceptors embedded in the skin above the thyroid cartilage is sufficient. A 40 Hz VTS was only capable of stimulating tactile mechanoreceptors in the skin, but not the deeper laryngeal mechanoreceptors. In contrast, a 100 Hz stimulation could potentially trigger responses of mechanoreceptors in laryngeal muscles, the mucosa of the epiglottis and sensory corpuscles at the free edge of the vocal cords (26, 27). Analysis indicated that both the 40 and 100 Hz stimulation can induce voice improvements in people with LD (see Figure 4). This is an important finding, because it means that tactile stimulation of the skin above the larynx can be sufficient to reduce voice symptoms of people with LD. This allows for the use of smaller vibrators with lower amplitude in future VTS wearable devices that could be used easily in every-day life (39).

### Interaction of botulinum neurotoxin injection with laryngeal VTS

Prior to this study, there was no knowledge of whether and how Botulinum toxin would affect possible treatment effects of VTS. Yet, addressing this question is important because approximately 50–60% of patients with adductor type of LD receive benefits from regular Botulinum injection as a symptomatic treatment (4). Participants treated with BoNT entered the study approximately 14 days after their last injection as many patients treated with BoNT have acute injection effects (e.g., whispering voice). For those who received BoNT, week 6 typically corresponded to the period, where voice symptoms were at their lowest point (i.e., their “golden period”), and week 11 was at the end of their BoNT cycle, when they became again symptomatic. The multiple baseline study design allowed to dissociate the effects of BoNT from possible VTS effects as the pre-VTS assessment at each study visit represented their current their voice status influenced by BoNT and the post-VTS indicated their voice symptoms influenced by BoNT plus laryngeal VTS (see Figures 1B,C). We assessed participants three times (week 1, 6, 11) and found that those participants who were treated with BoNT could receive an additional benefit from VTS at each time point (see Figure 4). Moreover, the markers of voice quality did not decline significantly in the BoNT group at week 11 as would be expected, if the positive changes in voice quality were solely determined by BoNT injection. Finally, those participants not treated with BoNT could also receive acute benefits from VTS (see Figure 4).

### Limitations and alternative explanations

To fully delineate the effects VTS in the presence or absence of BoNT, it needs to be recognized that the intramuscular application of BoNT is a peripheral intervention affecting both extrafusal muscle fibers as well as muscle spindles and, in addition, evokes plastic changes within the central sensorimotor system including cerebral cortex (40, 41). Thus, to gain a complete understanding of the interaction of BoNT with VTS, it would be desirable to stratify for BoNT and employ additional neurophysiological measures to elucidate, for example, differences in electrocortical responses between BoNT and non-BoNT users. The current study only provided initial evidence, but its design and scope were not able to address such a complex issue.

The duration of VTS effects tended to be short, which does limit its usefulness as a treatment. Given the small weekly dosage received in this study, we have no knowledge if higher dosages would increase the duration of effectiveness over time.

One needs to recognize that this study did not use the rainbow passage test sentences (42) that has been used to diagnose LD. We here followed newer guidelines of a recent consensus paper (1) recommending that LD research should use sentences loaded with voiced phonemes for diagnosis and monitoring treatment response. The rainbow passage is not loaded with voiced phonemes like the AdLD sentences by Ludlow et al. (2) used in this study.

Finally, at this point we have no clear understanding why some people with LD respond to VTS and others do not. It may be argued that the observed VTS-induced benefits partially or fully constitute an unspecific placebo effect. However, given that known electrocortical response to VTS in somatosensory-motor cortex occurs within the matter of seconds, and VTS yields improvements in voice symptoms in minutes (37), and given that this study documents that these acute improvements are repeatable over months, it seems very unlikely that VTS is an unspecific placebo.

## Conclusion

This study provides the first systematic empirical evidence that laryngeal VTS constitutes a non-invasive form of neuromodulation that can induce acute improvements in voice quality and reduces voice effort in people with adductor-type LD. The results suggest that low-frequency VTS targeting tactile mechanoreceptors of the skin above the larynx can be sufficient to temporarily reduce LD symptoms. An additional benefit of the approach is that VTS is low-cost and simple to administer, which makes it attractive and suitable as an in-home treatment.

## Data availability statement

The raw data supporting the conclusions of this article will be made available by the authors, without undue reservation.

## Ethics statement

This study involved humans and was approved by Institutional Review Board of the University of Minnesota - Twin Cities (STUDY00004618). It was conducted in accordance with the local legislation and institutional requirements. All participants provided their written informed consent prior to participation in this study.

## Author contributions

JK: Conceptualization, Funding acquisition, Investigation, Methodology, Project administration, Supervision, Visualization, Writing – original draft, Writing – review & editing. DB: Data curation, Investigation, Methodology, Project administration, Supervision, Writing – original draft, Writing – review & editing, Visualization. NE: Conceptualization, Methodology, Writing – original draft, Writing – review & editing. JO: Data curation, Formal analysis, Investigation, Visualization, Writing – original draft, Writing – review & editing. GG: Conceptualization, Resources, Writing – original draft, Writing – review & editing. PW: Conceptualization, Methodology, Writing – original draft, Writing – review & editing.

## Supplementary material

SUPPLEMENTARY FIGURE S1 Consort flow diagram.

SUPPLEMENTARY MATERIAL 2 Trial protocol.

SUPPLEMENTARY MATERIAL 2 CONSORT checklist.
