# Supplementary figures and images for: Effects of an 11-week vibro-tactile stimulation treatment on voice symptoms in laryngeal dystonia
Source: Front Neurol. 2024 May 30;15:1403050. doi: 10.3389/fneur.2024.1403050 (PMC11169659; doi:10.3389/fneur.2024.1403050)

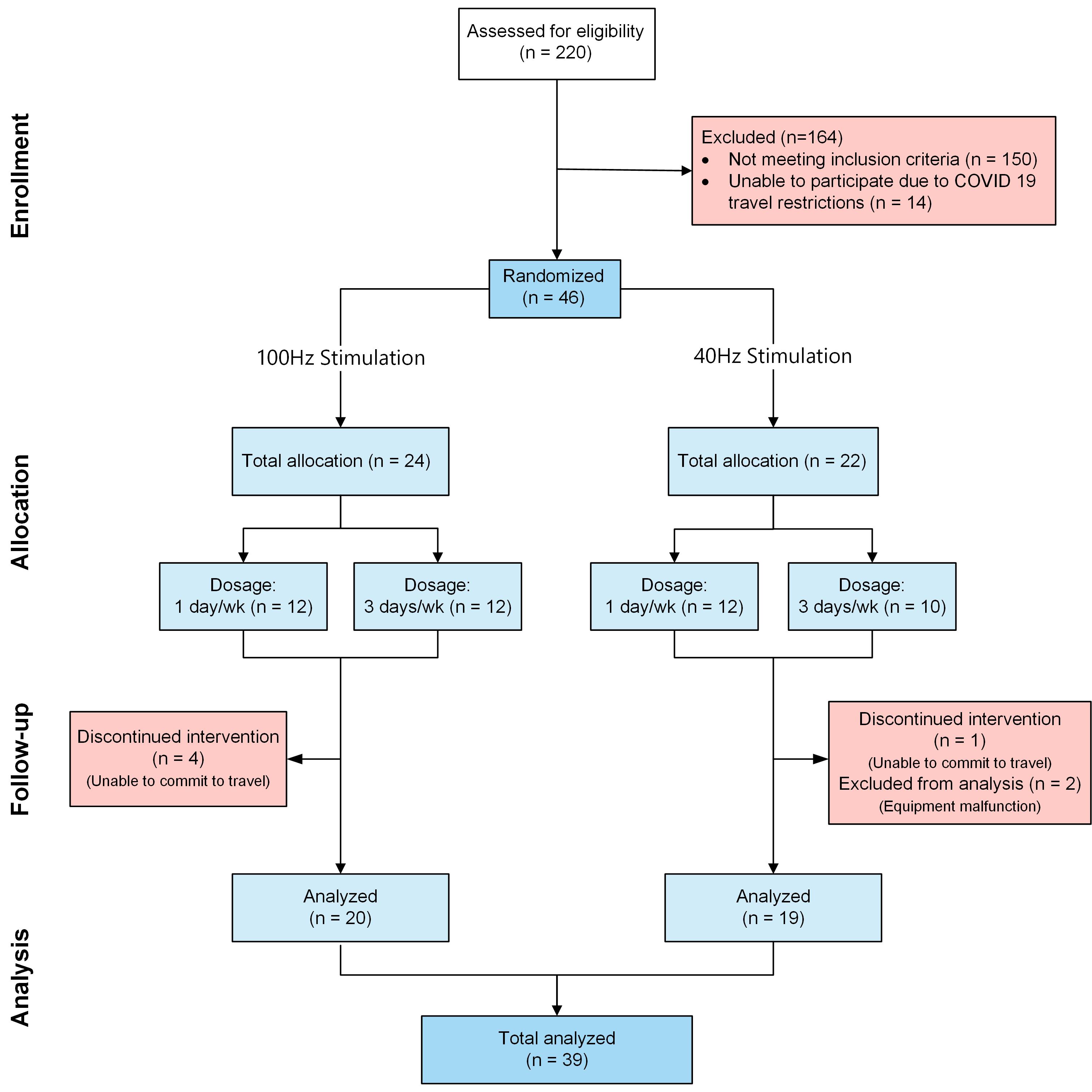

Supplement: SUPPLEMENTARY FIGURE S1 — Consort flow diagram. [file Image_1.JPEG]
