# Supplementary material for: Effects of an 11-week vibro-tactile stimulation treatment on voice symptoms in laryngeal dystonia
Source: Front Neurol. 2024 May 30;15:1403050. doi: 10.3389/fneur.2024.1403050 (PMC11169659; doi:10.3389/fneur.2024.1403050)
Supplement: SUPPLEMENTARY MATERIAL 2 — Trial protocol. [file Table_1.DOCX]

**PROTOCOL TITLE:**

Laryngeal vibration as a non-invasive treatment for spasmodic dysphonia

Table of Contents

[1.0 Objectives 6](#_Toc494449131)

[2.0 Background 6](#_Toc494449132)

[3.0 Study Endpoints/Events/Outcomes 8](#_Toc494449133)

[4.0 Study Intervention(s)/Investigational Agent(s) 9](#_Toc494449134)

[5.0 Procedures Involved 10](#_Toc494449135)

[6.0 Data and Specimen Banking 14](#_Toc494449136)

[7.0 Sharing of Results with Participants 14](#_Toc494449137)

[8.0 Study Population 14](#_Toc494449138)

[9.0 Vulnerable Populations 15](#_Toc494449139)

[10.0 Local Number of Participants 15](#_Toc494449140)

[11.0 Local Recruitment Methods 16](#_Toc494449141)

[12.0 Withdrawal of Participants 16](#_Toc494449142)

[13.0 Risks to Participants 17](#_Toc494449143)

[14.0 Potential Benefits to Participants 17](#_Toc494449144)

[15.0 Statistical Considerations 17](#_Toc494449145)

[16.0 Confidentiality 19](#_Toc494449146)

[17.0 Provisions to Monitor the Data to Ensure the Safety of Participants 19](#_Toc494449147)

[18.0 Provisions to Protect the Privacy Interests of Participants 19](#_Toc494449148)

[19.0 Compensation for Research-Related Injury 20](#_Toc494449149)

[20.0 Consent Process 20](#_Toc494449150)

[21.0 Setting 20](#_Toc494449151)

[22.0 Multi-Site Research 20](#_Toc494449152)

[23.0](#_Toc494449153) [References 21](#_Toc494449154)

**ABBREVIATIONS/DEFINITIONS**

- EEG: Electroencephalogram
- FD Focal dystonia
- SD: Spasmodic dysphonia
- VTS: Vibro-tactile stimulation

**STUDY SUMMARY**

| **Study Title** | Laryngeal vibration as a non-invasive treatment for spasmodic dysphonia (SD) |
| --- | --- |
| **Study Design** | A 2 x group (low /high frequency vibration treatment/) design with two subgroups (high/low dosage training). The high frequency SD group will receive effective vibro-tactile stimulation (VTS) at 100Hz frequency and the low frequency SD group will receive ineffective VTS at 40Hz stimulation frequency for a total training period of 8 weeks. Each group will be divided into two sub-groups – a low dosage and a high dosage training group. The subgroups will cross-over after 4 weeks of training. There is no healthy control group included in this protocol. |
| **Primary Objective** | Determine the effects of repeated vibro-tactile stimulation (VTS) of the larynx on objective markers of speech quality in healthy human volunteers and people with spasmodic dysphonia, a voice disorder affecting the laryngeal muscles. |
| **Secondary Objective(s)** | Elucidate the neurophysiological mechanism behind the effectiveness of VTS by demonstrating that a) SD is associated with abnormally increased levels of neuronal synchronization in the sensorimotor cortex, and b) that VTS can reduce sensorimotor cortical excitation in SD by desynchronizing motor cortical neuron activity. |
| **Research Intervention(s)/Investigational Agents** | Vibro-tactile stimulation of the superficial skin surface above the larynx (human voice box) |
| **Scientific Assessment** | Nationally-based, federal funding organizations |
| **IND/IDE # (if applicable)** | NSR IDE |
| **IND/IDE Holder** | N/A |
| **Investigational Drug Services # (if applicable)** | N/A |
| **Study Population** | People diagnosed with spasmodic dysphonia |

# Objectives

- 1. Purpose:

The general aim of the research is to provide scientific evidence that VTS represents a non-invasive form of neuromodulation that can induce measurable improvements in the speech of SD patients. This research addresses a clinical need to develop alternative or auxiliary treatments for a rare voice disorder with limited treatment options. A successful completion of the proposed work will be an important step in advancing laryngeal VTS as a therapeutic intervention for improving the voice symptoms in SD. Specifically, the scientific yield by achieving the specific aims is threefold: First, it will elucidate the unknown neurophysiological mechanism behind laryngeal VTS by documenting the neural changes associated with VTS. Second, it will establish that VTS can improve voice quality in SD. Third, by documenting that laryngeal VTS yields long-term benefits on voice quality in SD patients, it would provide a solid basis for a clinical trial that needs to address open questions on optimal dosage and duration of VTS-based voice therapy, the magnitude of the therapeutic effect across adductor and abductor SD and its long term efficacy.

# Background

- 1. Significance of Research Question/Purpose:

The general aim of the study is to provide scientific evidence that VTS represents a non-invasive form of neuromodulation that can induce measurable improvements in the speech of SD patients. This work addresses a clinical need to develop alternative or auxiliary treatments for a rare voice disorder with very limited treatment options. A successful completion of the proposed work will be an important step in advancing laryngeal VTS as a therapeutic intervention for improving the voice symptoms in SD.

- 1. Preliminary Data:

We conducted a series of preliminary investigations to assess the feasibility of applying VTS to the human larynx. First, we searched for suitable vibrators that could be applied to the skin above the voice box and provide the necessary amplitude to penetrate sufficiently the thyroid cartilage and to stimulate the underlying mucosal mechanoreceptors and/or proprioceptors of extrinsic laryngeal muscles. Second, we recorded the acoustic signals during vocalization with and without VTS to determine its effect on voice production. Third, we measured the EEG responses to laryngeal vibration to understand the cortical responses to laryngeal VTS.

Selecting and piloting appropriate vibrators. We systematically tested a range of commercially available, small precision vibrators that delivered amplitudes between 1.7-14.3 G (based on 100g inertial load testing). Vibrators were attached to the skin above the larynx of healthy volunteers and tested under varying voltage inputs. During testing subjects repeatedly vocalized vowel tones for 6-second intervals that were recorded for later acoustic frequency analysis. It became clear that vibrators with amplitudes above 6G are unsuitable (e.g. induced a gag reflex at higher voltage; uncomfortable to wear for prolonged periods of time (> 10 min). We systematically narrowed the selection to a encapsulated cylinder vibrator (Fig. 2) that has a maximum amplitude < 6G and produced clearly detectable periodic signals in the acoustic data at frequencies that were within the frequency range known to stimulate laryngeal mechanoreceptors [1].

Existing Literature:

SD is a characterized by involuntary, random movement of laryngeal muscles causing disruption of fluent speech with strained-strangled voice quality. SD is more prevalent in women [2, 3]. Onset is typically in midlife. There are two types of SD: (a) adductor (AD) typified by uncontrolled vocal fold closure, and (b) abductor (AB) characterized by uncontrolled vocal fold opening. The AD form is more common and typically occurs during the voiced components of speech. SD symptoms are task specific, occurring during speech but not during other phonatory (e.g., prolonging vowels) or non-phonatory tasks (e.g., breathing). SD shares several abnormal neurologic signs with FD of the head, neck and hand. For example, abnormal blink reflexes were observed in SD, torticollis, and blepharospasm [4-7] and abnormal long-latency responses to peripheral nerve stimulation have been observed in SD [8], in blepharospasm and oromandibular dystonia [9]. Recent evidence from our group and the work of others strongly indicate that basal ganglia-related diseases such as Parkinson’s disease or certain forms of dystonia are associated with somatosensory and specifically proprioceptive abnormalities that are closely linked to the observed motor deficits [10-21] (for reviews see: [22, 23]). Finally, our own work confirmed that SD as a voice disorder is associated with a deficit in arm kinaesthesia [24] – a finding consistent with results from patients with FD of the head and neck [21].

Neurophysiology behind VTS. It has long been established that VTS can stimulate muscle spindles [25-27] and mechanoreceptors [28] affecting motor behavior and inducing changes in kinaesthesia [29-31]. In general, vibrating the skin at amplitudes of ≤ 15 µm is sufficient to activate Ia muscle spindle aﬀerents of superficial muscles, which evokes a contractile response called the Tonic Vibration Reflex [26, 27]. To elicit kinaesthetic illusions, vibration must typically range between 40-100 Hz [32, 33]. Brief vibration to a relaxed muscle typically leads to an increase in muscle tone that can easily be overcome by voluntary phasic innervation [34], while excitatory input to spinal α-motor neurons is depressed when vibration is applied for prolonged periods [35]. At the cortical level it has been shown that prolonged muscle tendon vibration of wrist flexors (30 min) induced an increase in corticospinal excitability of the antagonistic wrist extensors lasting up to 60 min after vibration indicating that VTS can induce measurable changes in short-term cortical plasticity [36, 37].

Somatosensory deficits and VTS in FD. Numerous research reports documented somatosensory deficits in FD (for review see [38]). For example, proprioceptive-based finger position sense thresholds and the perception of arm motion are abnormal in patients with cervical dystonia or blepharospasm [21, 39]. The abnormalities in tactile and proprioceptive processing are not restricted to the affected dystonic musculature, but were also documented in non-affected body regions [21, 40, 41] indicating a generalized somatosensory deficit in FD. Recording of somatosensory evoked potentials (SEPs) and TMS data document that abnormal processing of somatosensory information in FD is associated with abnormally enhanced cortical excitability and decreased intracortical inhibition [42, 43], which also has been confirmed for SD [44]. The susceptibility of FD to somatosensory stimulation has long been known, because patients with task-specific dystonia may use sensory tricks (geste antagoniste) to temporarily alleviate dystonic symptoms by touching or pressing areas of or near the dystonic musculature [45, 46]. Research on cervical dystonia documented that effective sensory tricks are associated with pallidal and motor cortical desynchronization at low frequencies (6-8Hz) [47]. It has further been shown that FD responds to VTS. Vibrating dystonic neck muscles of patients with torticollis, who exhibit abnormally tilted head postures, induced head righting and nearly restored normal head posture [48]. Vibrating non-dystonic arm muscles in patients with cervical dystonia and blepharospasm skewed arm position sense to a greater extent than healthy controls [39]. In summary, there is overwhelming evidence that somatosensory processing is affected in many forms of FD (for reviews see [22, 38]). VTS has been shown to influence somatosensory perception and may reduce the severity of dystonic postures [13, 39, 45, 48].

One challenge in applying VTS to the speech motor system is that intrinsic laryngeal muscles and the mucosa of the epiglottis are shielded by thyroid cartilage. While proprioceptors of limb muscles can be stimulated by placing vibrators on the skin above them, this is not possible for laryngeal muscles. That is, non-invasive vibrators are needed that can be easily attached to the skin above the larynx and vibrate with sufficient amplitude to induce responses in laryngeal mechanoreceptors. Many of the vibrators that have been used in the past to investigate vibration responses in limb muscles are not suitable due to size, weight and voltage requirements. Fortunately, recent advancements in vibrator technology (largely driven by cell phone engineering) have produced small, low voltage, yet powerful vibrators that, for the first time, make it feasible to wear and operate vibrators at the neck for a prolonged time without restricting a person’s movement.

# Study Endpoints/Events/Outcomes

- 1. Primary Endpoint/Event/Outcome:

If successful, the work under this protocol would lay the scientific foundation for a clinical trial to examine the usefulness of the approach in a larger patent sample. It would document the sensorimotor cortical activation patterns associated with SD and the cortical responses to VTS. It would promote development of wearable, user-programmable medical devices that could apply VTS while monitoring its effect on voice production in real-time. Ultimately, VTS would enlarge the available therapeutic arsenal by either augmenting existing Botox therapy or becoming an alternative intervention option for patients who do not tolerate Botox injections.

# Study Intervention(s)/Investigational Agent(s)

- 1. Description:

The vibratory motors (Precision MicrodrivesTM, Model 307 – 100) used are low-voltage (~1V) and non-invasive. For this protocol, they vibrate at frequencies between 5 - 100 Hz (i.e. 100 times per second). The small electric motors are encapsulated (see **Fig. 1**). There are no moving parts that can come in contact with the skin. At a stimulation frequency of 100Hz, the inertial load is approximately 2.7G, which translates to a vibration amplitude well below the threshold that may induce a swallowing reflex (> 6G). The device is powered by a standard variable DC power supply with a maximum voltage output of 3V.

The device is labeled in accordance with the labeling provisions of the IDE regulations (§812.5) bears the statement "CAUTION - Investigational Device. Limited by Federal (or United States) law to investigational use."


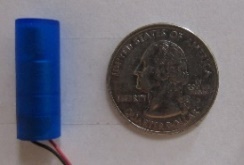


**Figure 1**. Vibratory motor used for this study (Precision Microdrives Ltd., London, UK, Model 307 – 100) Length: 25 mm. Diameter: 9 mm.

- 1. Drug/Device Handling:

Operation: The investigator or the patient (during in-home training) will attach the vibrators to the skin using a hypoallergenic surgical tape. When the vibrators are turned on, the strength of the vibration is similar to the vibration experienced when touching a vibrating cell phone or gaming joystick. Vibro-tactile stimulation at the applied frequency and amplitude is not known to cause pain or tissue damage. The participant may feel a mild tingling or vibrating sensation. Preliminary testing on healthy human subjects showed that at the given vibration parameters (see 4.1) no adverse reactions occur.


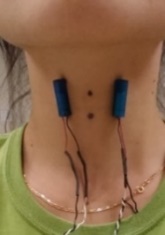


**Figure 2.** Placement of vibratory motors on the larynx.

Storage: When not in use, the device is stored in a small plastic container in the laboratory storage room that is accessible only to authorized lab personnel. Patient participants who received the device from the investigators for in-home use training (see Table 2 below), will be instructed to keep it out of the reach of children.

# Procedures Involved

- 1. Study Design:

We employ a 2 x group (high/low vibration frequency) design with two subgroups (high/low training dosage). The high frequency SD group will receive effective VTS at 100Hz frequency and the low frequency SD group will receive ineffective VTS at 40Hz stimulation frequency for a total training period of 8 weeks. Each group will be divided into two sub-groups – a low dosage and a high dosage training group. The subgroups will cross-over after 4 weeks of training. Because the optimal frequency of receiving VTS is unknown, we opt for a strategy that a) allows to discriminate between two training frequencies (low and high dosage mode), b) is modeled on our approach for aim 1, which had produced positive results based on our preliminary data, and c) is not too time-consuming, thus minimizing the barrier for participation and consequently guarding against attrition or non-compliance. There will be two follow up points (week 6 & 11) with lab testing in order to obtain longitudinal data on objective markers of voice quality. In addition, the EEG data collected during the follow-up visits will allow us to map changes in cortical activation due to laryngeal VTS, thus, providing a neural correlate for expected changes in voice quality (see **Table 2** for an overview of the design). All participants of the low vibration frequency group will get the opportunity to cross-over to the 100 Hz VTS treatment after the completion of the study (i.e. after the end of week 11; at the earliest in week 12). Participants of the low frequency group will be informed in the final follow-up meeting in week 11 of this opportunity. In the case that a participant of the low frequency group desires to this “cross-over”, the participant will keep the equipment for another 11 weeks and will be instructed by the investigators on how to set the vibrators to the 100 Hz frequency. In-home training for potential cross-over participants will follow the same protocol as specified in Table 2 (high-frequency group).

Low frequencytreatment. We opt to apply low frequency stimulation over no stimulation where the participant only receives tactile feedback. The reason for not selecting a no vibration/pure tactile stimulation sham treatment is that participants likely realize that this is a sham treatment, because the whole setup is in turned off mode. In addition, our own pilot data have shown that pure tactile stimulation with the vibrators turned off does not result in voice improvement. Moreover, stimulation frequencies < 40 Hz are generally ineffective in eliciting kinaesthetic illusions or tonic vibration effects [32, 33]. Finally, based on our own measures the signal attenuation for laryngeal VTS ranges approximately between 20-40Hz. That is, a 40Hz VTS will unlikely stimulate mechanoreceptors in the epiglottis or the laryngeal musculature.

**Table 2**. Experimental design for achieving aim 2. All participants will undergo the same in-laboratory assessments and standardized training described in detail in the approach for aims 1, 3 (see Fig 8.). In-home training will be either once-a-week (low frequency), or every-second-day (high frequency). Subgroups will cross over in week 6. High and low frequency groups (each N=16) are divided into two subgroups.

|  | Week 1 | Weeks 2-5 | Week 6 | Weeks 7-10 | Week 11 |
| --- | --- | --- | --- | --- | --- |
| Subgroups | *In-lab Training*  *Begin* | *In-home Training* | *In-lab Training 6-wk Follow Up* | *In-home Training* | *In-lab Training*  *Final Follow Up* |
| High Freq Group 1  (N=8) | Voice Ass. + Lab Training **100Hz VTS**  (effective) | **Low intensity**  (Once-a-week @ 100Hz) | Voice Ass. + Lab Training **100Hz VTS**  (effective) | **High intensity**  (Every-second-day @ 100Hz) | Voice Ass. + Lab Training **100Hz VTS**  (effective) |
| High Freq .  Group 2  (N=8) |  | **High intensity**  (Every-second-day @ 100Hz) |  | **Low intensity**  (Once-a-week @ 100Hz) |  |
| Low Freq Group 1  (N=8) | Voice Ass. + Lab Training  **40Hz VTS**  (ineffective) | **Low intensity ineffective**  (Once-a-week @ 40Hz) | Voice Ass. + Lab Training  **40Hz VTS**  (ineffective) | **High intensity ineffective**  (Every-second-day @ 40Hz) | Voice Ass. + Lab Training  **40Hz VTS**  (ineffective) |
| Low Freq Group 2  (N=8) |  | **High intensity ineffective**  (Every-second-day @ 40Hz) |  | **Low intensity ineffective**  (Once-a-week @ 40Hz) |  |

Randomization of sample. Patient assignment to the respective groups will be randomized to control for selection or accidental bias. We will employ a block/adaptive covariate randomization following [49, 50]. The block randomization ensures a balanced assignment of subjects across groups resulting in equal sample sizes for the four subgroups. The covariate adaptive randomization using the low/high dosage training condition as “covariate” ensures that any new participant will be sequentially assigned to one of the four groups by taking into account previous group assignments of participants (total N=32; N=8 per group). This randomization requires a sample size that is an even multiple of the number of blocks. Thus, the minimum is 2x4 blocks = 8 participants per subgroup. The cross-over design will allow for group comparisons at N=16 subjects. Both the subgroup and group sample size is greater than N=6 the number of subjects required per group to show significant change in voice quality as based on CPP (see power analysis in section Sample Size Considerations). A respective randomization plan has been generated.

- 1. Study Procedure: In-laboratory assessment and training

Subjects will perform voice production tasks before, during and after vibration. Two light-weight, low voltage, non-invasive surface electrical vibratory motors will be attached to the skin of the anterior portion of the neck adjacent to the voice box to vibrate the larynx (see **Fig. 2**). Voice production before, during, and after the vibration will be recorded via a microphone for subsequent acoustic analysis on voice quality by speech analysis experts. In addition, during voice production, electroencephalography (EEG) will be recorded with a 64-channel system (Active 2 BioSemi B.V., Amsterdam, The Netherlands) at a sampling frequency of 512 Hz. Subjects will wear a regular head cap arranged in the standard 10-20 configuration (see

**Figure 1**). Four additional external electrodes will be used. Two electrodes will be placed above and below the left eye detect eye movements. Another two electrodes will be attached on bilateral mastoid processes to serve as reference electrodes. A water-based conductive gel is applied between the electrodes and the skin, which can be cleaned easily with water.

#
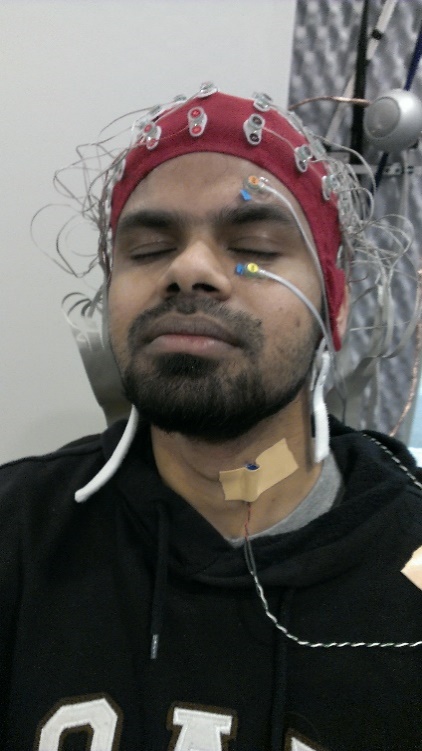


**Figure 1**. EEG cap with 10-20 electrode configuration

Voice assessment. To assess voice quality, participants will perform two voice production tasks. First, they will vocalize the vowels ‘ahh’ and ‘ee’. Second, they read aloud ten test sentences. They will perform each task three times per assessment. They will repeat these assessments several times during testing (see **Fig. 4**). The sentence reading task consists of a set of test sentences based on a 20-sentence inventory described by Ludlow et al. as being sensitive to the voice symptoms experienced in SD [2]. Participants will read the sentences at their own comfortable pace and loudness. In addition, participants will rate their perceived effort level of vocalization on an ordinal scale of 0 to 10 (0 being with no effort and 10 being with maximal effort).

Application of laryngeal vibration. Each VTS trial begins with a 10-second rest period, followed by 4 seconds of vocalization. After 2 seconds of vocalization, vibration will set in while the subject continues to vocalize. Thus, each trial has a *rest*, a *vocalization only*, and a *vocalization + vibration* period. Each participant performs 50 trials per set.

The timeline of the complete protocol is illustrated in Figure 4. Testing will begin with a pretest voice assessment, followed by three sets comprising the *vibration only* and *vocalization + vibration* conditions. The order of the conditions will be counter-balanced between subjects to account for possible order effects. During the *vibration only* condition, subjects will be silent and are allowed to read for diversion. After each set a 5-min break will be given and fluids will be offered. Voice assessment batteries will be administered after each set. After completion of all three sets, three final voice assessments follow immediately after treatment (Post 3 OFF), and 20 min and 60 min after VTS has stopped (Post 20, Post 60).

5 min

5 min

10 min

***Fig. 4****. Flow chart of voice assessment and in-lab training. Total exposure time to vibration will be 45 min. Each set will last 25 minutes. There will be 5 minutes breaks between sets. The interspersed, repeated voice assessment will allow to document time-dependent changes in voice quality and cortical activation due to laryngeal vibration up to 60 minutes past VTS application. Voice assessment pretest 1 is performed 60 minutes before pretest 2 (multiple baseline).*

**Set 1**

**Set 2**

**Set 3**

20 min

Voice Assess. **Pretest**

**1**

**Vocal.**

**+ VTS**

**VTS**

**Only**

Voice Assess. **Post 1**

**OFF**

Voice Assess. **Post 1**

**VTS ON**

**Vocal.**

**+ VTS.**

**VTS**

**Only**

Voice Assess. **Post 2**

**OFF**

Voice Assess. **Post 2**

**VTS ON**

**Vocal.**

**+ VTS.**

**VTS**

**Only**

Voice Assess. **Post 3**

**OFF**

Voice Assess. **Post 3**

**VTS ON**

40 min

Break

Voice Assess.

**Post 20**

Voice Assess. **Post 60**

**Retention**

5 min

5 min

Voice Assess. **Pretest**

**2**

**Baseline**

- 1. Study Duration of In-lab Assessment and Training:

The study consists of a single session. Total exposure time to vibration will be approximately 45 minutes. Each VTS set will last 25 minutes. There will be 5 minutes breaks between sets. The interspersed, repeated voice assessment will allow to document time-dependent changes in voice quality and cortical activation due to laryngeal vibration up to 60 minutes past VTS application. Total duration of the study will be 180 minutes.

- 1. Study Procedure: In-home-training

For each day of scheduled training (see **Table 2**), participants will apply VTS for a total duration of 20 minutes. With vibrators on, they will vocalize the vowels ‘ahh’ and ‘ee’ each five times and read aloud the 20 test sentences from Appendix A of Ludlow et al. [2]. They will then engage in spontaneous speech in response to a self-posed question: "How is my voice functioning today and do I recognize any benefits from training" (modeled after the CAPE-V questionnaire). Toward the end of practice, with the vibrators turned off, they will read aloud again the test sentences developed by Ludlow et al. [2]. They will record their voices using the voice recording application of their smartphone or digital tablet (if they do not own a digital recording device, we will supply an iPad tablet). These devices are simple to use and while the recording quality will not lend itself to rigorous acoustic analysis, the recordings will provide objective data on the number and duration of voice breaks and provide qualitative data on how participants experience the training. At the end of each training, they will email their voice records to the study coordinator, who will anonymize the records before submitting them to Drs. Watson and Urberg for further analysis. The approach to include an in-home training component is an efficient and pragmatic way to investigate possible long-term effects of VTS on the voice quality in SD.

5.4a Study Procedure: Optional remote audio testing

In case the follow up laboratory visits are not feasible, testing will be conducted remotely. The testing will be conducted via a video call. A high quality microphone will be provided and the participants will be asked to download a digital audio recording software. Participants will then be asked to vocalize the vowels ‘ahh’ and ‘ee’ each five times and read aloud the 20 test sentences from Appendix A of Ludlow et al. [2]. Participants will read the sentences at their own comfortable pace and loudness. In addition, participants will rate their perceived effort level of vocalization on an ordinal scale of 0 to 10 (0 being with no effort and 10 being with maximal effort). Participants will apply the laryngeal vibration on themselves as instructed for a period of 10 minutes. The voice production tasks will be performed before vibration, during vibration and after vibration. The application of laryngeal vibration and voice assessment will be the same as the in-laboratory visit.

- 1. Study Duration of In-home Training:

Duration of in-home training is 8 weeks. See **Table 2** for an overview of when in-home training will take place during the study.

- 1. Study Duration of Complete Study:

Total study duration will be a maximum of 11 weeks. See **Table 2** for an overview.

- 1. Individually Identifiable Health Information:

For SD participants, we collect health information concerning disease duration, and any possible treatment (Botox injection) that they receive and when the last Botox injection had occurred prior to testing. The respective HIPAA Agreement Template Form has been uploaded to ETHOS. The PI and his study team have no direct access to any other medical records*.*

# Data and Specimen Banking

N/A.

# Sharing of Results with Participants

N/A

# Study Population

- 1. Inclusion Criteria:

Patients: Diagnosis of adductor spasmodic dysphonia for a minimum of 6 months with documented symptom relief after Botox injection. Diagnosis is made by a voice disorder specialist. There is a higher prevalence for women in SD with a female/male ratios reported between 2.85:1 to 4.1:1 [2, 51]. For 2016, the female/male ratio of botox treated SD patients at the UM Lion’s Voice Clinic was 74:29, i.e. 2.55:1 (Mean age: 61.8 yrs. ± 10.8 Std). Based on these ratios, we expect to recruit approximately 65% female patients (26 females, 14 males).

- 1. Exclusion Criteria:

Patients:

1. Regular intake of benzodiazepines
2. Cognitive impairment: score < 27 on Mini-mental state examination
3. Identifies with a neurological or musculoskeletal impairment affecting speech motor function. These impairments may include a form of: Dyskinesia, Dystonia, Essential Tremor, Huntington’s Disease, Multiple System Atrophy, Muscle Tension Dysphonia, Parkinsonism, Progressive Supranuclear Palsy, Spasticity, Intracranial Neoplasm (brain tumor), Spinal Neoplasm, Cerebrovascular Accident (Stroke), Mild Traumatic Brain Injury, Intracranial Hemorrhage, Multiple Sclerosis
4. Abductor spasmodic dysphonia (ABSD). Although our preliminary data show that ABSD may benefit from VTS, we justify the exclusion of ABSD patients, because the ratio of ADSD/ABSD is approximately 9:1 [51], which means we could, at best, only recruit a small sample of ABSD patients in this single-center study.

Screening: SD participants will be screened by Doctors at the Fairview Lion’s Voice Clinic. All SD subjects will be assessed by the clinicians using an established protocol [2]. The clinical assessment consists of a questionnaire (CAPE V) [52], clinical speech evaluation and a nasoendoscopic exam. These clinical assessments are part of the routine exam that patients receive for diagnostic purposes. They are not an explicit part of this study.

Controls: No healthy controls will be tested under this protocol.

# Vulnerable Populations

- 1. Vulnerable Populations:

Children

Pregnant women/Fetuses/Neonates

Prisoners

Adults lacking capacity to consent and/or adults with diminished capacity to consent, including, but not limited to, those with acute medical conditions, psychiatric disorders, neurologic disorders, developmental disorders, and behavioral disorders

Approached for participation in research during a stressful situation such as emergency room setting, childbirth (labor), etc.

Disadvantaged in the distribution of social goods and services such as income, housing, or healthcare

Serious health condition for which there are no satisfactory standard treatments

Fear of negative consequences for not participating in the research (e.g. institutionalization, deportation, disclosure of stigmatizing behavior)

Any other circumstance/dynamic that could increase vulnerability to coercion or exploitation that might influence consent to research or decision to continue in research

Undervalued or disenfranchised social group

Members of the military

Non-English speakers

Those unable to read (illiterate)

Employees of the researcher

Students of the researcher

None of the above

# Local Number of Participants

- 1. Local Number of Participants to be Consented:

Number of patients with spasmodic dysphonia (SD) will be 40.

# Local Recruitment Methods

- 1. Recruitment Process:

Voice-disordered subjects (SD) will be recruited through our own SD patient database, through Doctors at the Fairview Lion’s Voice Clinic, MN.

Identification of Potential Participants:

SD patients are seen regularly by Doctors and Otolaryngologists as part of their clinical routine. Dr.______ sees approximately 30-40 patients a month and administers Botox injections as medically indicated. Both clinicians will identify potential participants and inform them about the study possibility. The initial contact to potential participants is made by doctors at their respective clinics. We will not access patient databases in either clinic to identify potential participants through the study coordinator. Note that potential participants are regularly seen by Doctors as part of the ongoing patient treatment (e.g. Botox injections). At these scheduled visits Doctors will share the study information flyer (see ETHOS section: Recruitment Materials) with potential participants and explain to them the scope of the study. If patients are interested in participating, the contact information of the study coordinator will be shared with the patient, who can then contact the study team. Once the study coordinator is contacted by a potential participant, he will explain the study procedure in detail and will invite participation. If agreeable, a date for obtaining informed consent and data collection is set. Prior to study begin investigators will obtain and document informed consent from each participant according to[21 CFR 50](https://www.accessdata.fda.gov/scripts/cdrh/cfdocs/cfCFR/CFRSearch.cfm?CFRPart=50), Protection of Human Subjects (see also section 20.0).

Doctors at Fairview Clinic have legitimate access to patient PHI as part of their clinical work. Relevant PHI (type of diagnosis, disease duration, frequency of Botox injections) will not be shared with the study team of the PI before consent has been obtained and the participant has signed the appropriate HIPAA form.

- 1. Recruitment Materials:

SD participants will be invited to the study via flyers that summarize the study and its goals.

- 1. Payment:

Study participants will be reimbursed $50 for each in-lab study visit (max. $150 for three visits).

# Withdrawal of Participants

- 1. Withdrawal Circumstances:

Participants may opt to take a break or withdraw at any time during the in-lab study procedure, if they experience discomfort or become fatigued.

Although our preliminary investigation showed that the applied vibration frequency and amplitude is not sufficient to induce a swallowing or gag reflex, we will stop the procedure, if such incidence is observed. Elicitation of a swallowing reflex is not harmful, but its repeated non-volitional elicitation may cause discomfort.

- 1. Withdrawal Procedures:

If such event would occur, we would give the participant the option to adjust the vibration parameters or to completely withdraw from the study. Any data collected up to that point would be destroyed and not used in further analysis.

- 1. Termination Procedures:

Upon reaching the limit of the proposed study population, recruitment will stop and only data analysis will continue. Once the analysis is completed, the de-identified data sets will remain on UMN HIPAA compliant servers. They will form the basis for any scientific publication resulting from the study. We will then orderly terminate the study following the procedures required by IRB.

# Risks to Participants

- 1. Foreseeable Risks:

Vibration Risks. The risks in this study are minimal other than the possibility of skin redness over the skin of the voice box or feelings mimicking numbness over the neck area during trials. These discomforts, if any occurs, usually disappear within minutes after vibration is removed.

EEG Risks. There is no particular risk associated with EEG recordings. The procedure is non-invasive and no energy is imposed by the system to the participant. Participants may opt to take a break or stop/withdraw at any time, if they experience any discomfort due the wearing of the EEG cap.

# Potential Benefits to Participants

- 1. Potential Benefits:

There is no direct benefit to participating in this study. At this point, there are no known data that provide conclusive evidence for the effectiveness of laryngeal vibration in reducing voice disorder symptoms.

# Statistical Considerations

- 1. Data Analysis Plan:

Analysis of voice data. Acoustic analysis will follow the methodology described by [53]. Waveform and wide-band spectrograms will be used to identify and measure the duration of the variables. The acoustic signal will be examined for the presence of phonatory breaks and aperiodic breaks. Phonatory breaks are defined as any voiced phonemic segment without sound that is greater than 50ms; the duration of phonatory breaks will be measured. Aperiodic segments will be identified as any voiced phonemic segment that contains non-repetitive cycles that are greater than 50ms; and the duration of the break will be measured. To standardize the duration measures for both phonatory and aperiodic breaks, the sum of the duration of breaks will be divided by the sum of the duration of all voiced segments for each subject. Inter-rater reliability will be measured using Cohen’s alpha. Intra-rater reliability will be established using Pearson’s r correlation (expected to range between r=.89 to r= .98 according to [53]). For both break types, the absence of phonation or the presence of aperiodicity at the boundary of a syllable or the beginning of a word will not be considered. We will perform a MANCOVA procedure employing all acoustic variables with pre-/posttests as independent factors. If the MANCOVA is significant, separate univariate ANOVAs will be performed on each acoustic variable (dependent) and assessment conditions as a repeated measure (independent) [53]. Appropriate Bonferroni adjustments will account for multiple testing.

Analysis of EEG data. EEG recordings during speech are known to be susceptible to noise from the multiple muscles in and near the head that are involved in speech production. We have been cognizant to this fact and a) designed a simple vocalization task that does not involve complex patterns of muscle activation and b) use modern noise removal techniques that have been successfully applied for the analysis of voice related EEG. Dr. Yang is an expert in the analysis of speech-related EEG and will lend his expertise for EEG data analysis, which is based on the widely utilized MATLAB EEGlab toolbox [54]. Space limitation does not permit to describe all processing steps in detail. However, here we outline its major approach. First, to remove motion and electromyographic artifacts, all channels will be filtered (0.5 Hz high-pass and 20 Hz low-pass FIR filter). Second, target events will be extracted and trials containing non-physiological signal amplitudes will be rejected (typically outside [-100 μv 100 μv]). Third, for further objective noise removal the ADJUST algorithm (Automatic EEG artifact Detector based on the Joint Use of Spatial and Temporal features) will be utilized - algorithm also included in the NIH Neuroimaging Tools and Resources Platform. Finally, an Independent Component Analysis (ICA) will be applied [55]. The ICA statistically decomposes all 64 channels into 64 independent sources of information (components) whose weighted sum is assumed to result in the formation of the recorded data. After this step, the pruned components are combined, generating a cleaner data set. After the ICA weights have been computed, the ADJUST will be employed to detect those independent components, which based on the statistical criteria of the algorithm, were the potential sources of stereotyped artifacts (eye blinks, vertical eye movement, horizontal eye movement, generic discontinuity i.e. inappropriate signals due to noisy channels or muscle activation) [56]. The statistical criteria for removing noise components include temporal kurtosis and spatial average difference [SAD] (from eye blinking), the maximum epoch variance [MEV] and SAD (from vertical eye movements), the MEV and spatial eye difference [SED] (from horizontal eye movements), and MEV and the generic discontinuity spatial feature [GDSF] (from bad channels and muscle activation). After the removal of noise components, a time-frequency analysis will be carried out on all channels of the pruned dataset. The analysis will yield the mean event-related changes in the spectral power “ERSP” (from pre-stimulus baseline) for the different frequency ranges at each time instance of all epochs.

# Confidentiality

- 1. Data Security:

All private information will be stored in a secured location and all recorded and derived data will be de-identified and stored separately on a password protected University of Minnesota HIPAA compliant server. In any publications or presentations, we will not include any information that will make it possible to identify a subject. However, a participant record for the study may be reviewed by departments at the University with appropriate regulatory oversight.

- 1. Media and Publication Authorization Form:

All participants are allowed to participate voluntarily in the preparation of media materials. Should participants volunteer to participate, they will be required to complete a media and publication authorization form. The purpose of this form is to provide the participants’ authorization to release and use identifiable information about them, their specific condition and the treatment they received. The information may be used to prepare and publish education and training materials, case reports, videos, presentations, professional/medical publications, professional and staff communications, newspaper or other form of articles, broadcast stories, television newscasts, newsletters, advertising, brochures, websites, social or other media communications, marketing and fundraising materials and/or promotional materials (together referred to as “Media Materials”).

# Provisions to Monitor the Data to Ensure the Safety of Participants

- 1. Data Integrity Monitoring.

The PI and study coordinator oversee the progress of the study and to ensure that it is conducted, recorded, and reported in accordance with the protocol, standard operating procedures, and applicable regulatory requirements. Integrity of data will be monitored regularly by the PI and the study coordinator. This study will be listed with clinicaltrails.gov.

- 1. Data Safety Monitoring.

Protection of subject confidentiality will be strictly monitored. All records will be kept confidential and without identifying the subjects in published materials. Only authorized personnel directly associated with the study will have access to digital files or paper data sheets with original data sets that may contain information about subject identity. The paper files will be kept in locked file cabinets in the laboratory of the PI to which only authorized personnel has access. The files containing the subject identifying codes will be destroyed after 10 years. All digital data will be stored on a secure, HIPAA compliant server maintained by the University of Minnesota Office of Information Technology and/or using “Box” as the HIPAA compliant tool (see https://it.umn.edu/center-excellence-hipaa-data). All research personnel completed the required data and safety monitoring courses (e.g., responsible conduct of research, HIPAA, etc.).

# Provisions to Protect the Privacy Interests of Participants

- 1. Protecting Privacy: As described in section 11, participants will only provide contact information as it is needed to interact with them prior to or after testing. The collected PHI is limited and will only be used in de-identified form during analysis and in any possible scientific publications. During the consent process, we explicitly tell the participant what PHI, if any, will be collected and how long we will store it. We also describe the study procedure in detail and emphasize that the procedure is not invasive and carries no known health risk.
  2. Access to Participants*:*

Only the study coordinator has access to the PHI or address information of the participants. The other members of the study team only have access to the de-identfied data.

# Compensation for Research-Related Injury

- 1. Compensation for Research-Related Injury:

In the unlikely event that this research activity results in an injury, treatment will be available, including first aid, emergency treatment and follow-up care as needed. Care for such injuries will be billed in the ordinary manner to subjects or their insurance company.

# Consent Process

- 1. Consent Process (when consent will be obtained):
- Consent will take place in the laboratory prior to testing. Investigators will obtain and document informed consent from each participant according to[21 CFR 50](https://www.accessdata.fda.gov/scripts/cdrh/cfdocs/cfCFR/CFRSearch.cfm?CFRPart=50), Protection of Human Subjects.
- There are, at minimum, several days and often weeks between informing the prospective participants and obtaining the consent. This study requires that patients are seen towards the end of their Botox cycle. Once a potential study participant contacts the study coordinator, has received the relevant study information, and has agreed to participate, the coordinator and the participant agree on a date suggested by the participant. The patients know best when they become symptomatic and the effect of the Botox injection is wearing off.
- The referring clinician determines that a potential participant understands the information.
- Copies of the consent forms have been uploaded to ETHOS.

# Setting

- 1. Research Sites:

Locations where participants will be recruited. SD individuals will be invited through doctors at the Fairview Lion’s Voice Clinic.

Locations where the research will be conducted: University of Minnesota

# Multi-Site Research

N/A

# References

1. Davis, P.J. and B.S. Nail, *Quantitative analysis of laryngeal mechanosensitivity in the cat and rabbit.* J Physiol, 1987. **388**: p. 467-85.

2. Ludlow, C.L., et al., *Research priorities in spasmodic dysphonia.* Otolaryngology - Head and Neck Surgery, 2008. **139**(4): p. 495-505.e1.

3. Soland, V.L., K.P. Bhatia, and C.D. Marsden, *Sex prevalence of focal dystonias.* J Neurol Neurosurg Psychiatry, 1996. **60**(2): p. 204-5.

4. Cohen, L.G., et al., *Blink reflex excitability recovery curves in patients with spasmodic dysphonia.* Neurology, 1989. **39**(4): p. 572.

5. Tolosa, E. and L. Montserrat, *Depressed blink reflex habituation in dystonic blepharospasam.* Neurology, 1985. **35**(supplement 1): p. 251-260.

6. Tolosa, E., L. Montserrat, and A. Bayes, *Blink reflex studies in focal dystonias: enhanced excitability of brainstem interneurons in cranial dystonia and spasmodic torticollis.* Movement Disorders: Official Journal of the Movement Disorder Society, 1988. **3**(1): p. 61-69.

7. Topka, H. and M. Hallett, *Perioral reflexes in orofacial dyskinesia and spasmodic dysphonia.* Muscle & Nerve, 1992. **15**(9): p. 1016-1022.

8. Ludlow, C.L., et al., *Abnormalities in long latency responses to superior laryngeal nerve stimulation in adductor spasmodic dysphonia.* The Annals of Otology, Rhinology, and Laryngology, 1995. **104**(12): p. 928-935.

9. Berardelli, A., et al., *Pathophysiology of blepharospasm and oromandibular dystonia.* Brain: A Journal of Neurology, 1985. **108 ( Pt 3)**: p. 593-608.

10. Adamovich, S.V., et al., *The interaction of visual and proprioceptive inputs in pointing to actual and remembered targets in Parkinson's disease.* Neuroscience, 2001. **104**(4): p. 1027-1041.

11. Contreras-Vidal, J.L. and D.R. Gold, *Dynamic estimation of hand position is abnormal in Parkinson's disease.* Parkinsonism & Related Disorders, 2004. **10**(8): p. 501-506.

12. Demirci, M., et al., *A mismatch between kinesthetic and visual perception in Parkinson's disease.* Annals of Neurology, 1997. **41**(6): p. 781-788.

13. Konczak, J. and G. Abbruzzese, *Focal dystonia in musicians: linking motor symptoms to somatosensory dysfunction.* Front Hum Neurosci, 2013. **7**: p. 297.

14. Konczak, J., et al., *The perception of passive motion in Parkinson's disease.* Journal of Neurology, 2007. **254**(5): p. 655-663.

15. Konczak, J., et al., *Haptic perception of object curvature in Parkinson's Disease.* PLoS ONE, 2008. **3**(7): p. e2625.

16. Konczak, J., et al., *Parkinson's disease accelerates age-related decline in haptic perception by altering somatosensory integration.* Brain, 2012. **135**(Pt 11): p. 3371-9.

17. Maschke, M., et al., *Dysfunction of the basal ganglia, but not the cerebellum, impairs kinaesthesia.* Brain, 2003. **126**(Pt 10): p. 2312-22.

18. Maschke, M., et al., *Perception of heaviness in Parkinson's disease.* Movement Disorders: Official Journal of the Movement Disorder Society, 2006. **21**(7): p. 1013-1018.

19. Maschke, M., et al., *The effect of subthalamic nucleus stimulation on kinaesthesia in Parkinson’s disease.* Journal of Neurology, Neurosurgery & Psychiatry, 2005. **76**(4): p. 569 -571.

20. O'Suilleabhain, P., J. Bullard, and R.B. Dewey, *Proprioception in Parkinson's disease is acutely depressed by dopaminergic medications.* Journal of Neurology, Neurosurgery & Psychiatry, 2001. **71**(5): p. 607 -610.

21. Putzki, N., et al., *Kinesthesia is impaired in focal dystonia.* Movement Disorders, 2006. **21**(6): p. 754-760.

22. Patel, N., J. Jankovic, and M. Hallett, *Sensory aspects of movement disorders.* Lancet Neurol, 2014. **13**(1): p. 100-12.

23. Konczak, J., et al., *Proprioception and motor control in Parkinson's disease.* J Mot Behav, 2009. **41**(6): p. 543-52.

24. Chen, Y.W., P. Watson, and J. Konczak, *Reduced arm kinaesthetic sensitivity in spasmodic dysphonia.* Society for Neuroscience Abstracts, 2013. **43**: p. 437.17.

25. Brown, M.C., I. Engberg, and P.B. Matthews, *The relative sensitivity to vibration of muscle receptors of the cat.* J Physiol, 1967. **192**(3): p. 773-800.

26. Brown, M.C., I. Enberg, and P.B. Matthews, *The use of vibration as a selective repetitive stimulus for Ia afferent fibres.* J Physiol, 1967. **191**(1): p. 31P-32P.

27. Bianconi, R. and J. van der Meulen, *The response to vibration of the end organs of mammalian muscle spindles.* J Neurophysiol, 1963. **26**: p. 177-90.

28. Vedel, J.P. and J.P. Roll, *Response to pressure and vibration of slowly adapting cutaneous mechanoreceptors in the human foot.* Neurosci Lett, 1982. **34**(3): p. 289-94.

29. Goodwin, G.M., D.I. McCloskey, and P.B. Matthews, *Proprioceptive illusions induced by muscle vibration: contribution by muscle spindles to perception?* Science, 1972. **175**(4028): p. 1382-4.

30. Goodwin, G.M., D.I. McCloskey, and P.B. Matthews, *The contribution of muscle afferents to kinaesthesia shown by vibration induced illusions of movement and by the effects of paralysing joint afferents.* Brain, 1972. **95**(4): p. 705-48.

31. Roll, J.P. and J.P. Vedel, *Kinaesthetic role of muscle afferents in man, studied by tendon vibration and microneurography.* Exp Brain Res, 1982. **47**(2): p. 177-90.

32. Cordo, P., et al., *Proprioceptive consequences of tendon vibration during movement.* J Neurophysiol, 1995. **74**(4): p. 1675-88.

33. Cordo, P.J., et al., *Effect of slow, small movement on the vibration-evoked kinesthetic illusion.* Exp Brain Res, 2005. **167**(3): p. 324-34.

34. De Gail, P., J.W. Lance, and P.D. Neilson, *Differential effects on tonic and phasic reflex mechanisms produced by vibration of muscles in man.* J Neurol Neurosurg Psychiatry, 1966. **29**(1): p. 1-11.

35. Shinohara, M., *Effects of prolonged vibration on motor unit activity and motor performance.* Med Sci Sports Exerc, 2005. **37**(12): p. 2120-5.

36. Steyvers, M., et al., *Frequency-dependent effects of muscle tendon vibration on corticospinal excitability: a TMS study.* Exp Brain Res, 2003. **151**(1): p. 9-14.

37. Forner-Cordero, A., et al., *Changes in corticomotor excitability following prolonged muscle tendon vibration.* Behav Brain Res, 2008. **190**(1): p. 41-9.

38. Stamelou, M., et al., *The non-motor syndrome of primary dystonia: clinical and pathophysiological implications.* Brain, 2012. **135**(Pt 6): p. 1668-81.

39. Grünewald, R.A., et al., *Idiopathic focal dystonia: a disorder of muscle spindle afferent processing?* Brain, 1997. **120 (Pt 12)**: p. 2179-85.

40. Fiorio, M., et al., *Tactile temporal discrimination in patients with blepharospasm.* J Neurol Neurosurg Psychiatry, 2008. **79**(7): p. 796-8.

41. Molloy, F.M., et al., *Abnormalities of spatial discrimination in focal and generalized dystonia.* Brain, 2003. **126**(Pt 10): p. 2175-82.

42. Kanovsky, P., et al., *Abnormalities of cortical excitability and cortical inhibition in cervical dystonia Evidence from somatosensory evoked potentials and paired transcranial magnetic stimulation recordings.* J Neurol, 2003. **250**(1): p. 42-50.

43. Zeuner, K.E. and F.M. Molloy, *Abnormal reorganization in focal hand dystonia--sensory and motor training programs to retrain cortical function.* NeuroRehabilitation, 2008. **23**(1): p. 43-53.

44. Samargia, S., R. Schmidt, and T.J. Kimberley, *Shortened cortical silent period in adductor spasmodic dysphonia: Evidence for widespread cortical excitability.* Neurosci Lett, 2014. **560**: p. 12-5.

45. Kägi, G., et al., *Sensory tricks in primary cervical dystonia depend on visuotactile temporal discrimination.* Mov Disord, 2013. **28**(3): p. 356-361

46. Poisson, A., et al., *History of the 'geste antagoniste' sign in cervical dystonia.* J Neurol, 2012. **259**(8): p. 1580-4.

47. Tang, J.K., et al., *Changes in cortical and pallidal oscillatory activity during the execution of a sensory trick in patients with cervical dystonia.* Exp Neurol, 2007. **204**(2): p. 845-8.

48. Karnath, H., J. Konczak, and J. Dichgans, *Effect of prolonged neck muscle vibration on lateral head tilt in severe spasmodic torticollis.* Journal of Neurology, Neurosurgery & Psychiatry, 2000. **69**(5): p. 658-660.

49. Suresh, K., *An overview of randomization techniques: An unbiased assessment of outcome in clinical research.* J Hum Reprod Sci, 2011. **4**(1): p. 8-11.

50. Wichmann, B.A. and I.D. Hill, *An Efficient and Portable Pseudo-Random Number Generator.* Applied Statistics-Journal of the Royal Statistical Society Series C, 1982. **31**(2): p. 188-190.

51. Adler, C.H., B.W. Edwards, and S.F. Bansberg, *Female predominance in spasmodic dysphonia.* J Neurol Neurosurg Psychiatry, 1997. **63**(5): p. 688.

52. Zraick, R.I., et al., *Establishing validity of the Consensus Auditory-Perceptual Evaluation of Voice (CAPE-V).* Am J Speech Lang Pathol, 2011. **20**(1): p. 14-22.

53. Sapienza, C.M., S. Walton, and T. Murry, *Adductor spasmodic dysphonia and muscular tension dysphonia: Acoustic analysis of sustained phonation and reading.* Journal of Voice, 2000. **14**(4): p. 502-520.

54. Delorme, A. and S. Makeig, *EEGLAB: an open source toolbox for analysis of single-trial EEG dynamics including independent component analysis.* J Neurosci Methods, 2004. **134**(1): p. 9-21.

55. Hyvarinen, A. and E. Oja, *Independent component analysis: algorithms and applications.* Neural Netw, 2000. **13**(4-5): p. 411-30.

56. Mognon, A., et al., *ADJUST: an automatic EEG artifact detector based on the joint use of spatial and temporal features.* Psychophysiology, 2010. **48**(229-240).
